# Pushing the boundaries of few-shot learning for low-data drug discovery with a Bayesian meta-learning hypernetwork framework

**DOI:** 10.1093/bib/bbaf408

**Published:** 2025-08-15

**Authors:** Jiacai Yi, Dejun Jiang, Chengkun Wu, Xiaochen Zhang, Weixing He, Wentao Zhao, Dongsheng Cao

**Affiliations:** College of Computer Science and Technology, National University of Defense Technology, Deya Road, Changsha, Hunan 410073, PR China; Xiangya School of Pharmaceutical Sciences, Central South University, Tongzipo Road, Changsha, Hunan 410013, PR China; College of Computer Science and Technology, National University of Defense Technology, Deya Road, Changsha, Hunan 410073, PR China; Science and Technology on Parallel and Distributed Processing Laboratory, Deya Road, National University of Defense Technology, Changsha, Hunan 410073, PR China; Information Technology Department, Shangqiu Normal University, Pingyuan Road, Shangqiu, Henan 476000, PR China; The First Hospital of Hunan University of Chinese Medicine, Shaoshan Middle Road, Changsha, Hunan 410007, PR China; College of Computer Science and Technology, National University of Defense Technology, Deya Road, Changsha, Hunan 410073, PR China; Xiangya School of Pharmaceutical Sciences, Central South University, Tongzipo Road, Changsha, Hunan 410013, PR China

**Keywords:** meta-learning, few-shot learning, hypernetwork framework, Bayesian learning

## Abstract

Hunting for candidate compounds with favorable pharmacological, toxicological, and pharmacokinetic properties in drug discovery is essentially a low-data problem, as data acquisition is both challenging and costly. This inherent data limitation clashes with the requirements of many powerful deep learning models, which typically require large datasets. Here, we present Meta-Mol, a novel few-shot learning framework based on Bayesian Model-Agnostic Meta-Learning. Meta-Mol introduces a novel atom-bond graph isomorphism encoder that captures molecular structure information at the atomic and bond levels. This representation is further enhanced by a Bayesian meta-learning strategy, allowing for task-specific parameter adaptation and reducing overfitting risks. Additionally, a hypernetwork is employed to dynamically adjust weight updates across tasks, facilitating more complex posterior estimation. Our results demonstrate that Meta-Mol significantly outperforms existing models on several benchmarks, providing a robust solution to address data scarcity in drug discovery.

## Introduction

The drug discovery process is both costly and time-consuming, primarily due to the necessity of rigorous multi-stage trials to confirm the safety and efficacy of novel compounds. Early-stage prediction of a compound’s properties—such as absorption, distribution, metabolism, excretion, and toxicity, alongside physicochemical characteristics—is pivotal, as it substantially mitigates the risk of late-stage attrition [1, 2]. However, the data-intensive nature of conventional deep learning models poses a significant challenge in drug discovery, where datasets are frequently limited, particularly for rare and emerging diseases [3–5]. This conflict between the data-hungry nature of deep learning algorithms and the low-data problem prevalent in drug discovery hinders the application of these powerful models [6–8].

**Figure 1:**
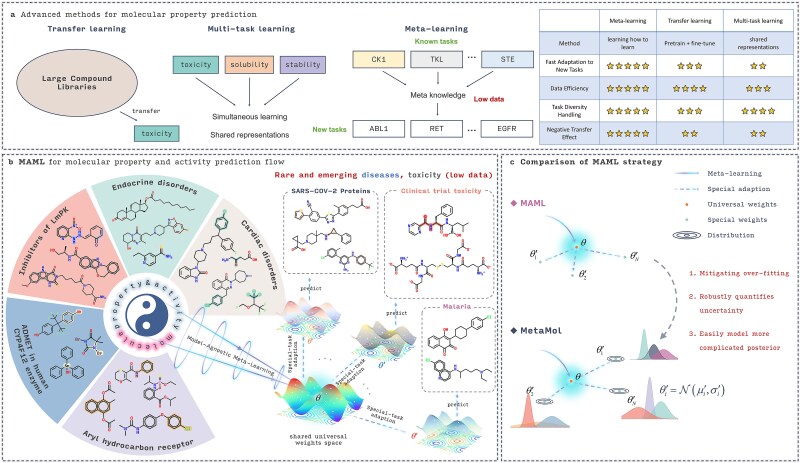
Conceptualization of Meta-Mol. (a) Advanced methods for molecular property prediction. (b) The MAML workflow in molecular property and activity prediction involves a process where the model learns a set of meta-parameters representing various related tasks based on existing ones and then rapidly adapts to new, unseen tasks using only a small amount of data. (c) Unlike previous approaches, Meta-Mol learns the universal weights point-wise, but a probabilistic structure is added when adapted for specific tasks.

Recently, advancements in methods such as transfer learning [9, 10] and multi-task learning [11, 12] have yielded promising results for molecular property prediction. Transfer learning typically leverages large compound libraries, where models are pretrained on related tasks and then fine-tuned for specific predictions, such as toxicity [13–15]. While effective for closely related tasks, this approach can suffer from negative transfer when task similarity is low [16]. Multi-task learning addresses this by simultaneously learning several related tasks, such as toxicity, solubility, and stability, through shared representations [17–19]. However, when encountering a new task, the model often requires retraining alongside existing tasks, which can be computationally expensive and time-consuming. Additionally, when data are sparse or imbalanced, the performance of multi-task learning may be hindered, as the model might struggle to generalize across tasks with limited or unevenly distributed data [20]. In contrast, meta-learning has emerged as a powerful approach for tackling data scarcity, a common challenge in molecular property prediction. Unlike traditional methods, meta-learning emphasizes learning to learn, enabling models to rapidly adapt to new tasks with minimal data. This method excels in scenarios with high task diversity, where only a small amount of data is available for each task. For example, as depicted in Fig. 1a, meta-learning can be particularly useful in kinase prediction, where the meta-knowledge acquired from predicting known kinases (e.g. CK1, TKL, STE) enables the model to effectively generalize to mutant kinases (e.g. ABL1, RET, EGFR) with minimal fine-tuning. This results in significantly improved data efficiency, enhanced ability to handle diverse tasks, and a reduction in negative transfer effects, making it more robust compared to conventional approaches like transfer learning and multi-task learning.

Model-Agnostic Meta-Learning (MAML) is one of the most popular and elegant few-shot learning methods. The core idea, as shown in Fig. 1b, is to learn shared universal weights within a meta-model, which can then be rapidly adapted for specific tasks, such as SARS-CoV-2 target prediction [21]. These shared universal weights represent a high-level abstraction of the relationship between molecular properties or activities and their structures. Nearly all MAML-based methods employ a bi-level optimization strategy. The outer loop adheres to the MAML framework for training, while the inner loop model, referred to as the inner model, is typically not strictly constrained [22]. Instead, it is chosen based on the task to find a model with better inductive biases. This flexibility is one of the key advantages of the MAML architecture, prompting several critical research questions: (i) How can outer-loop optimization be refined to better capture intertask relationships or adapt to task variations? (ii) How can the inner loop more precisely represent molecular data, where subtle structural variations may yield opposing properties, requiring effective modeling of both local atomic environments and nonlocal interactions among topologically distant atoms? (iii) How do data sampling strategies influence the efficacy of model learning?

There exist a few MAML-based methods for molecular properties prediction. MAML [22], Meta-MGNN [23], PAR [24], HSL-RG [25], CHEF [26], and Meta-GAT [27] typically learn meta-knowledge through gradient optimization, which is then updated to per-task local point-wise weights. Despite these methods achieving promising results, several major issues remain, including overfitting and insufficient uncertainty quantification. The limited information in small datasets, combined with the shift in their distribution compared to the ground truth distribution, weakens the model’s generalization capability [28, 29].

To overcome these issues, we consider the aforementioned three key aspects and propose a new meta-learning framework, Meta-Mol, which aims to mitigate data scarcity and model overfitting through graph models and Bayesian meta-learning (Fig. 1c). Specifically, (i) for outer-loop optimization, we introduce a Bayesian framework to learn a probabilistic structure rather than point-wise weights when adapting the model to new tasks. (ii) For the inner-loop model, we utilize a graph isomorphism network (GIN) to encode molecular information more effectively. Additionally, we implement dual encoding for atoms and chemical bonds to better capture local structural features. (ii) To counteract the effects of imbalanced data distributions, we propose a dynamic sampling strategy during the meta-learning training process. (iv) In this framework, task-specific weights are adjusted via a hypernetwork, replacing gradient-based optimization to enable modeling of complex structures and bolster adaptability. Through these advancements, Meta-Mol seeks to elevate the performance of molecular property prediction in low-data regimes, offering a potent solution for drug discovery challenges.

## Materials and methods

### An overview of the Meta-Mol framework

**Figure 2:**
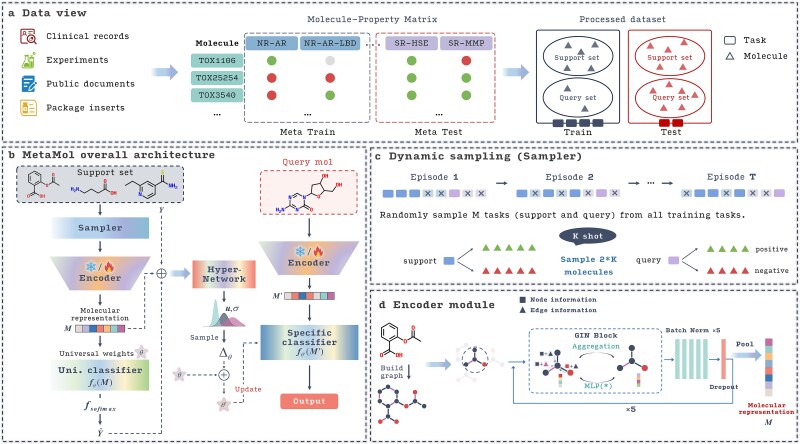
Main architecture of Meta-Mol and Submodule Diagram. (a) Example of data representation for meta-learning-based molecular property prediction. (b) The overall architecture of Meta-Mol. (c) Diagram of dynamic sampling. Squares with different patterns represent tasks in the support set and query set, respectively. (d) Structure encoder module.

Following the general MAML framework, Meta-Mol is designed as a two-level meta-learning workflow to address molecular property prediction in few-shot scenarios, as illustrated in Fig. 2b. It learns general patterns between molecules and their properties, represented by model weights $\theta$, which are universal shared parameters across tasks. During the testing, the model learns task-specific weights ${\theta}^{\prime }$ through a few iterations starting from the initial parameters $\theta$, with these tasks typically having only a few samples. In our model, a Bayesian MAML variant is developed to learn the point-wise general weights $\theta$ (referred to as “universal weights” in Fig. 2b) and the task-specific posterior distribution to obtain ${\theta}_i^{\prime }$. We assume that ${\theta}_i^{\prime }$ are independent, with a prior distribution $p\left({\theta}_i^{\prime}\right)=\mathcal{N}\left({\theta}^{\prime }|0,\mathrm{I}\right)$. Instead of traditional Bayesian methods that might infer posteriors through complex backpropagation rules on the task-specific parameters, we introduce a hypernetwork ${H}_{\Omega}$ (the “Hyper-Network” module in Fig. 2b) to directly learn and output the parameters (e.g. mean and variance) of the posterior distribution, for the weights of the “specific classifier.” The model can theoretically fit any posterior distribution with a sufficiently expressive hypernetwork. This generalization not only simplifies the complex modeling process of Bayesian MAML but also allows for more complex posterior distributions. The hypernetwork ${H}_{\Omega}$ effectively acts as a generator of task-specific adaptation parameters for the classifier, taking information from the support set of a new task and producing the instructions (mean and variance of a Gaussian distribution) on how to adjust the universal weights $\theta$ of the predictor to become task-specific ${\theta}_i^{\prime }$. This allows the “specific classifier” to be rapidly specialized for each new task. A brief description of an example is as follows. According to the concept of episodes [22], during the meta-training phase, the sampler dynamically samples Ns tasks from the preprocessed support set, with 2K molecules for each task. The sampled molecules [e.g. aspirin, “O=C(C)Oc1ccccc1C(=O)O”] are constructed into molecular graphs and fed into the encoder, which is pretrained on the ChEMBL dataset using a pretraining strategy guided by the principles from Hu *et al*. to learn molecular structural paradigms [30]. The molecular representations output by the encoder, denoted as $M$, are passed through a classifier to obtain prediction results. The classifier’s weights are subsequently subjected to Gaussian perturbations, which depend on the hypernetwork to adapt to new samples. The concatenation of the molecular representations, predicted probabilities, and true labels is fed into the hypernetwork ${H}_{\Omega}$, which outputs a normal probability structure of the model’s weight sets for the specific classifier, represented by means and variances. During the query phase, weight deltas are randomly sampled from the previously obtained Gaussian distribution using the reparameterization technique and applied to the universal weights of the target classifier network, thereby enabling Bayesian inference. (Fig. 2b).

### Encoding and prediction modules

Meta-Mol is composed of four modules: Structure Encoder, Sampler, Task-specific Predictor, and Hypernetwork, as illustrated in Fig. 2. Structure Encoder (Fig. 2d) is designed to encode molecular environmental information for the subsequent prediction task. Sampler dynamically selects subsets of molecules to create support and query sets for each task, facilitating the meta-learning process. Task-specific Predictor takes the embeddings generated by the Structure Encoder and, with the help of weights provided by the Hypernetwork, makes predictions tailored to specific molecular property tasks. Hypernetwork generates task-specific adjustments to the model’s parameters, enabling rapid adaptation to new tasks. To address overfitting in low-data scenarios, we adopted a meta-learning paradigm based on a Bayesian framework. This approach uses hypernetwork-based weight updates rather than gradient-based methods, capturing complex posterior distributions and enhancing task adaptability.

Following the MAML learning process, Meta-Mol learns general meta-knowledge across multiple known tasks and fine-tunes with a few samples to predict new molecular properties in novel tasks. During the meta-training phase, in the e-th episode, Meta-Mol learns meta-knowledge across different tasks using a bi-level training approach, where both inner and outer loops are employed on tasks sampled by the Sampler from the task pool. Without loss of generality, for a given task ${T}_i=\left\{{S}_i,{Q}_i\right\}$, Meta-Mol ${F}_{\theta }$ iterates over the support set ${S}_i$ and computes the loss on the query set ${Q}_i$ to perform gradient descent. ${F}_{\theta }$ consists of a molecular structure encoder $E\left(\ast \right)$ and a predictor ${H}_{\theta }$. The universal weights $\theta =\left({\theta}^E,{\theta}^H\right)$ include ${\theta}^E$ for the structure extractor and ${\theta}^H$ for the predictor. During a single forward pass, the data flows through both modules to perform the prediction:

(1) Specifically, for the Structure Encoder, we employ a message-passing mechanism [31] combined with a graph isomorphism network to encode the structural information of molecules (Fig. 2d). In addition to atomic features, we also incorporate bond information (see Table S1). This is achieved by designing an edge predictor that estimates bond connections during each iteration: 


(1) \begin{equation*} {h}_{\nu}^{(k)}= ML{P}^{(k)}\left({h}_{\nu}^{\left(k-1\right)}+\sum_{u\in \mathcal{N}\left(\nu \right)}\left({h}_u^{\left(k-1\right)}+{e}_{\nu u}\right)\right) \end{equation*}

(2) \begin{equation*} {\displaystyle \begin{array}{c}{h}_v^{(0)}={f}_{atom}(v)+{f}_{chir}(v)\end{array}} \end{equation*}

(3) \begin{equation*} {\displaystyle \begin{array}{c}{e}_{vu}={f}_{bond}\left({e}_{vu}^{type}\right)+{f}_{dir}\left({e}_{vu}^{dir}\right)\end{array}} \end{equation*}

where ${MLP}^{(k)}$ is a multi-layer perceptron, ${h}_v^{\left(k-1\right)}$ represents the feature vector of the neighboring node $u$ from the previous layer, and ${e}_{vu}$ is the edge feature between nodes $v$ and $u$, which is computed by embedding the bond type ${f}_{bond}\left({e}_{vu}^{type}\right)$ and bond direction ${f}_{dir}\left({e}_{vu}^{dir}\right)$. Each node $v$ in the graph is initialized with a feature vector ${h}_v^{(0)}$, which is derived from the embedded atomic ${f}_{atom}(v)$ and chirality information ${f}_{chir}(v)$.

After $K$ layers of message passing, the node features are aggregated to produce a graph-level representation using a pooling function: 


(4) \begin{equation*} {\displaystyle \begin{array}{c}{h}_G= AGG\left(\left\{{h}_v|v\in G\right\}\right)\end{array}} \end{equation*}

Here, ${h}_G$ represents the molecule-level feature, encapsulating the aggregate structural and feature information of the entire graph. The aggregation function $AGG$ can be flexibly defined to capture different aspects of the graph’s structure and properties, depending on the specific task requirements.

(2) With the molecule-level feature ${h}_G$ obtained, Meta-Mol then leverages a property predictor ${H}_{\theta }$ to perform the final molecular property prediction. The predictor is designed as a fully connected neural network that takes the aggregated graph-level representation ${h}_G$ as input and outputs the predicted property values: 


(5) \begin{equation*} {\displaystyle \begin{array}{c}{z}_{i+1}= ReLU\left({W}_i{z}_i+{b}_i\right)\end{array}} \end{equation*}

(6) \begin{equation*} {\displaystyle \begin{array}{c}\hat{y}={W}_l{z}_L+{b}_L\end{array}} \end{equation*}

where ${z}_i$ is the output of the *i*-th layer and $\hat{y}$ represents the predicted probability of each class.

### Bayesian hypernetwork module

When meta-training in a task ${T}_i=\left\{{S}_i,{Q}_i\right\}$, the parameters $\theta$ are updated to ${\theta}_i^{\prime }$. In the previous MAML method, such an update is achieved in one or more gradient descent updates on ${T}_i$ (inner loop). In the simplest case of one gradient update, the parameters are updated as follows: 


(7) \begin{equation*} {\displaystyle \begin{array}{c}{\theta}_i^{\prime }=\theta -\alpha{\nabla}_{\theta }{\mathcal{L}}_{T_i}\left({f}_{\theta}\right)\end{array}} \end{equation*}

where $\alpha$ is a step size hyperparameter used to control the magnitude of the gradient update. After completing the inner loop update, the model computes the loss on the query set ${Q}_i$ and uses these losses to update the model’s initial parameters $\theta$ (outer loop):


(8) \begin{equation*} {\displaystyle \begin{array}{c}\theta \leftarrow \theta -\beta{\nabla}_{\theta}\sum_{T_i\sim p(T)}{\mathcal{L}}_{T_i}\left({f}_{\theta_i^{\prime }}\right)\end{array}} \end{equation*}

where $\beta$ is a step size hyperparameter in the meta-learning process. This process uses the loss on the query set to optimize the model’s initialization parameters, enabling the model to better adapt to new tasks.

In our framework, Meta-Mol uses nongradient-based updates generated by hypernetworks (Fig. 2b). The hypernetwork ${H}_{\Omega}$​ takes as input the support set embeddings and produces the posterior distributions of the model’s weights. This allows the model to sample from these distributions to obtain different sets of weights, which helps capture the uncertainty inherent in the task. When adapting a function ${f}_{\theta }$ with parameters $\theta$ to a task ${T}_i$, the update process in a Bayesian hypernetwork can be described as: 


(9) \begin{equation*} {\displaystyle \begin{array}{c}{\theta}_i^{\prime}\sim q\left(\theta |{\lambda}_i\Big(\theta, {S}_i\right)\Big)\end{array}} \end{equation*}

(10) \begin{equation*} {\displaystyle \begin{array}{c}{\lambda}_i\left(\theta, {S}_i\right):= {H}_{\Omega}\left(\theta, {S}_i\right)\end{array}} \end{equation*}

where $q$ represents the posterior distribution of the model parameters given the support set ${S}_i$ and the hypernetwork ${H}_{\Omega}$. Additionally, the hypernetwork $H$ has a fixed number of parameters, regardless of how many tasks ${T}_i$ are used for training. In our model, the posterior is modeled as a Gaussian distribution, parameterized by a mean $u$ and the logarithm of the variance $logvar$: 


(11) \begin{equation*} {\displaystyle \begin{array}{c}\left({u}_{\theta}\left({S}_i\right),{logvar}_{\theta}\left({S}_i\right)\right):= {H}_{\Omega}\left(\theta, {S}_i\right)\end{array}} \end{equation*}

(12) \begin{equation*} {\displaystyle \begin{array}{c}{\theta}_i^{\prime}\sim \mathcal{N}\left\{\theta +{u}_{\theta}\left({S}_i\right),\exp \left({logvar}_{\theta}\left({S}_i\right)\right)\right\}\end{array}} \end{equation*}

Specifically, to construct the input for the hypernetwork ${H}_{\Omega}$, the embeddings ${E}_S$ are first generated from the support set $S$ using the encoder $E\left(\ast \right)$. These embeddings are then concatenated with their corresponding class labels ${Y}_S$ and the predicted values $\hat{Y_S}$ obtained from the general model ${f}_{\theta}\left({E}_S\right)$, forming a comprehensive input matrix. This concatenated matrix forms the input to the hypernetwork, which allows the hypernetwork to directly assess the general model’s current performance on the support set, enabling it to generate more informed, task-specific adjustments to the weights aimed at correcting observed errors and improving adaptation. The hypernetwork, composed of fully connected layers with ReLU activations, subsequently outputs the parameters that control the posterior distribution.

Finally, we approximate the learning objective using mini-batches as: 


(13) \begin{align*} &{\mathcal{L}}_T=\sum_{T_i\sim p(T)}\left[\frac{1}{P}{\sum}_{\theta_i^{\prime}\sim q\left(\theta, {\lambda}_i\left(\theta, {S}_i\right)\right)}\left[{\mathcal{L}}_{T_i}\left({f}_{\theta_i^{\prime }}\right)\right.\right.\nonumber\\&\quad\quad\quad\quad-\gamma KL\left(q\right({\theta}_i^{\prime }|\theta, {\lambda}_i\left(\theta, {S}_i\right)\left)\parallel \mathcal{N}\right({\theta}_i^{\prime }|0,\mathrm{I}\left)\right)\Big]\Big] \end{align*}

The detailed pseudocode for the Meta-Mol model, illustrating the complete training and adaptation process, can be found in Table S2.

### Data preparation

For all datasets from the MoleculeNet benchmark, we adopted the data processing approach outlined by Zhuang *et al* [32]. Specifically, for the PubChem BioAssay (PCBA) dataset, we selected the first five and the last five properties as the meta-test set, with the remaining properties used for meta-training. For the other datasets, we used the final *N* properties as the meta-test set, with detailed splits provided in Tables S3 and S4. Additionally, for all molecules, we performed Kekulé conversion to standardize their aromatic structures. We also precomputed atomic and bond information, which was stored for subsequent use in the model. The computed descriptors and features are listed in Table S1. Figure 2 illustrates the data workflow within the Meta-Mol execution process. Data from various sources are collected and organized into a unified dataset, typically formatted as a structured molecule-property matrix, where each entry indicates the presence or absence of specific molecular properties across different assays. In our example, green and red dots indicate labels 0 and 1, respectively. To align with the meta-learning framework, the model divides the dataset into nonoverlapping meta-training and meta-testing tasks. Finally, for both the inner and outer loops, the appropriate molecules for each task are sampled into support and query sets, ensuring no overlap between them.

### Dynamic sampling

In the training process of the meta-learning module, we construct an episode for each specific task in each outer loop, following the strategy proposed by Finn *et al* [22]. We introduced a dynamic sampling method during the meta-learner training process, where the support set and query set for each property task will be reselected randomly in each training epoch, as shown in Fig. 2c. Compared to static sampling strategies where tasks and their corresponding support/query splits are fixed, our dynamic approach continually exposes the model to varied data configurations within each task. This enhances robustness and prevents the model from overfitting to specific support-query compositions seen during meta-training. In the K-shot scenario, K positive and K negative samples are drawn from the support set in each iteration. This method helps to mitigate the effect of an unbalanced data distribution on model training [33], and it can better learn the relationships between different tasks by dynamically and randomly setting different scenarios.

### Training

We used PyTorch and PyTorch Geometric to build the model. RDKit was used to process the molecules. The Adam optimizer with an initial learning rate of $1\times{10}^{-3}$ and a multi-step learning rate schedule with a decay of 0.3 was used to train the model. The training was conducted over a total of 2500 epochs, with 20 episodes of learning iterations performed in each epoch. In each episode, we randomly sample five tasks from the task pool for meta-training. The hyperparameter settings for the model training process are detailed in Table S5. We ran experiments 10 times with different random seeds and report the mean and standard deviations. The average area under the receiver operating characteristic curve (AUROC) was calculated and reported in the Results and Discussion section. All experiments were conducted on an Ubuntu Server equipped with a single NVIDIA GeForce RTX Graphics Processing Unit (GPU) with 24 GB of memory.

## Results and Discussion

### Meta-Mol outperforms existing methods across various datasets

We first evaluated the few-shot prediction performance of Meta-Mol on several foundational datasets, demonstrating its advantages over other baseline models and methods. For this evaluation, we utilized widely recognized datasets in MoleculeNet benchmark [34]: Tox21 [35], SIDER [36], Maximum Unbiased Validation (MUV) [37], PCBA [38], and ToxCast [39]. Detailed descriptions of the datasets can be found in Supplementary Note 1 and Table S3. The ROC-AUC score was used as the evaluation metric for comparison. Each experiment was run 20 times with different random seeds to obtain reliable and robust model results. We conducted tests in both 10-shot and 1-shot scenarios, reporting the mean and standard deviation of the ROC-AUC scores across all meta-test tasks.

We used the same approach as the public splits for Tox21, SIDER, and MUV to divide the tasks into training and testing sets [40]. As for the PCBA dataset, we chose the first five and last five properties as meta-testing and the rest of properties as meta-training. For ToxCast, we first grouped its properties according to assay providers, resulting in nine subsets to obtain denser datasets. After discarding subsets with few properties, each remaining subset was divided into meta-training and meta-testing sets. Detailed information for each subset is provided in Table S4.

In this scenario, we compared Meta-Mol with four types of baselines: (1) classical few-shot method, including Siamese [41], ProtoNet [42], IterRefLSTM [40], EGNN [43], and TPN [44]; (2) meta-learning approaches with learning from scratch, including MAML [22], MAML-MetaMix [45], and Sharp-MAML [46]; (3) single-task methods, including Random Forest (RF) [47] and GraphConv [48]; and (4) graph-based molecular encoders utilizing pretraining, including Meta-MGNN [23], Pre-GNN [30], and Pre-PAR [49]. Table 1 presents the performance of different methods. The results of baseline methods are acquired from published studies [24, 25]. Across five datasets, Meta-Mol achieved competitive results compared to other baseline methods, outperforming the best baseline (Pre-PAR) in 8 out of 10 outcomes. On average, Meta-Mol showed a 5.9% improvement over Pre-PAR for each dataset. According to the statistics, MAML-based methods demonstrate superior performance compared to traditional few-shot learning methods in the current molecular property prediction task. This can be attributed to the advantages of the MAML architecture, which optimizes for generalization across different tasks by learning universal meta-knowledge rather than being tailored to specific tasks. This makes it more adaptable and robust. Additionally, methods based on molecular graph pretraining, such as Pre-PAR, Pre-GNN, and Meta-Mol, generally outperform traditional methods. This is intuitive, as these models learn molecular structure patterns from large datasets during the pretraining phase, which aids in further learning the relationships between molecular structures and their properties [50]. We observed a significant improvement of Meta-Mol on the SIDER dataset. We believe that this is because the SIDER dataset does not have missing labels and contains a relatively small amount of data. Compared to other methods, Meta-Mol, which uses a Bayesian framework, effectively mitigates overfitting and better extracts valuable information from the data. Additionally, Meta-Mol achieved satisfactory results on the Tox21, ToxCast, and PCBA datasets despite the missing labels, which could potentially cause uncertainty or bias during the model’s training process. However, we observed that severe label loss can still introduce significant bias, leading to a decline in model performance. For instance, in the MUV dataset, only 15.76% of the labels are available.

**Table 1:** The ROC-AUC scores ($\boldsymbol\uparrow $) on five benchmark datasets of molecular property prediction, compared with the classical few-shot method (first group), meta-learning approaches with learning from scratch (second group), single-task methods (third group), and methods that leverage pretrained molecular encoder (fourth group).

**Method**	**Tox21**		**SIDER**		**MUV**		**ToxCast**		**PCBA**	
	1-shot	10-shot	1-shot	10-shot	1-shot	10-shot	1-shot	10-shot	1-shot	10-shot
Siamese	65.00 ± 1.58	80.40 ± 0.35	51.43 ± 3.31	71.10 ± 4.32	50.00 ± 0.17	59.96 ± 5.13	–	–	–	–
ProtoNet	65.58 ± 1.72	74.98 ± 0.32	57.50 ± 2.34	64.54 ± 0.89	58.31 ± 3.18	65.88 ± 4.11	56.36 ± 1.54	63.70 ± 1.26	55.79 ± 1.45	64.93 ± 1.94
IterRefLSTM	80.97 ± 0.10	81.10 ± 0.17	71.73 ± 0.14	69.63 ± 0.31	48.54 ± 3.12	49.56 ± 5.12	–	–	–	–
EGNN	79.44 ± 0.22	81.21 ± 0.16	70.79 ± 0.95	72.87 ± 0.73	62.18 ± 1.76	65.20 ± 2.08	61.02 ± 1.94	63.65 ± 1.57	62.14 ± 1.58	69.92 ± 1.85
TPN	60.16 ± 1.18	76.05 ± 0.24	62.90 ± 1.38	67.84 ± 0.95	50.00 ± 0.51	65.22 ± 5.82	50.01 ± 0.05	62.74 ± 1.45	–	–
MAML	75.74 ± 0.48	80.21 ± 0.24	67.81 ± 1.12	70.43 ± 0.76	60.51 ± 3.12	63.90 ± 2.28	65.97 ± 5.04	66.79 ± 0.85	62.04 ± 1.73	66.22 ± 1.31
MAML-MetaMix	81.13 ± 0.21	81.83 ± 0.15	72.69 ± 0.31	73.34 ± 0.18	66.39 ± 1.22	67.38 ± 1.03	70.03 ± 1.14	70.22 ± 0.92	–	–
Sharp-MAML	74.59 ± 0.56	75.37 ± 0.23	68.43 ± 0.96	71.02 ± 0.81	65.12 ± 2.98	65.52 ± 2.01	66.49 ± 1.98	67.56 ± 1.01	–	–
RF	53.50 ± 0.06	58.60 ± 0.06	50.60 ± 0.04	53.50 ± 0.04	55.90 ± 0.10	58.40 ± 0.06	–	–	–	–
GraphConv	58.90 ± 0.07	64.80 ± 0.03	46.80 ± 0.05	48.30 ± 0.03	55.20 ± 0.08	56.80 ± 0.09	–	–	–	–
Meta-MGNN	82.13 ± 0.13	82.97 ± 0.10	73.36 ± 0.32	75.43 ± 0.21	65.54 ± 2.13	68.99 ± 1.84	–	–	–	–
Pre-GNN	81.68 ± 0.09	82.14 ± 0.08	73.24 ± 0.12	73.96 ± 0.08	64.51 ± 1.45	67.14 ± 1.58	72.90 ± 0.84	73.68 ± 0.74	–	–
Pre-PAR	83.01 ± 0.09	84.93 ± 0.11	74.46 ± 0.29	78.08 ± 0.16	$\boldsymbol{ 66.94}\,\pm\, \boldsymbol{1.12} $	$ \boldsymbol{69.96}\,\pm\,\boldsymbol{1.37} $	73.63 ± 1.00	75.12 ± 0.84	73.71 ± 0.61	72.49 ± 0.61
Meta-Mol	$ \boldsymbol{85.40}\,\pm\,\boldsymbol{0.50} $	$ \boldsymbol{86.21}\,\pm\,\boldsymbol{0.35} $	$ \boldsymbol{83.45}\,\pm\,\boldsymbol{0.90} $	**85.53 ± 0.89**	66.56 ± 0.84	69.55 ± 0.26	$ \boldsymbol{82.13}\,\pm\,\boldsymbol{0.68} $	$ \boldsymbol{84.48}\,\pm\,\boldsymbol{0.54} $	$ \boldsymbol{77.80}\,\pm\,\boldsymbol{0.85} $	$ \boldsymbol{79.13}\,\pm\,\boldsymbol{0.63} $

Note: “–” indicates data not available or not applicable.

**Figure 3:**
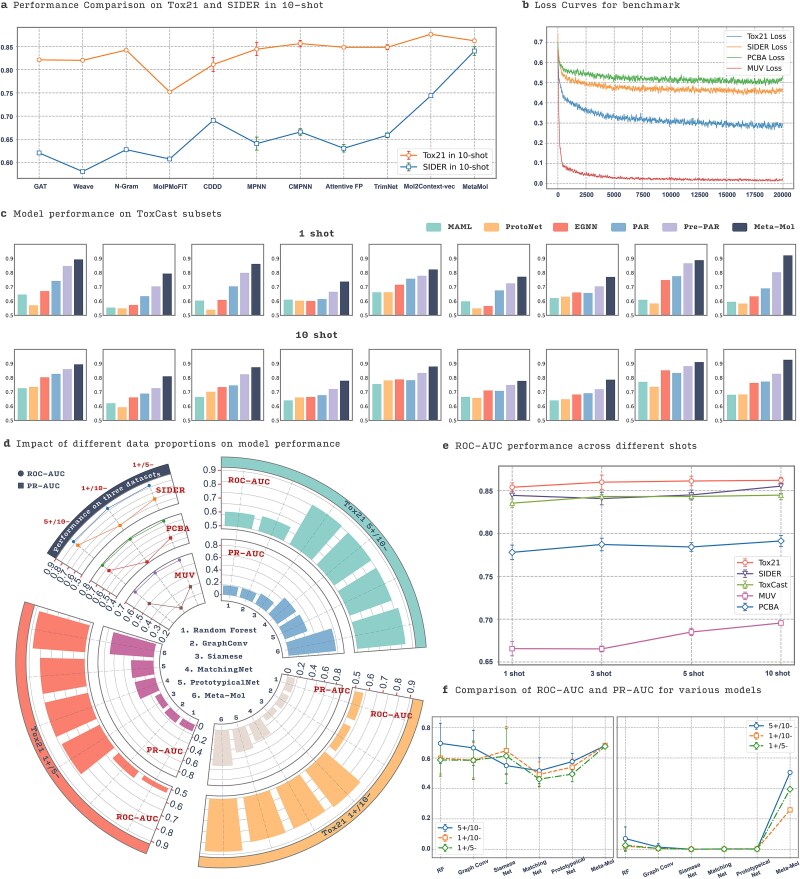
Comparative performance analysis diagram. (a) Performance evaluation of Meta-Mol and other representative methods on Tox21 and SIDER in 10-shot. (b) Loss curves for benchmark. The original data have been smoothed using a simple moving average with a window size of 40 to provide a clearer trend. (c) Model performance on ToxCast subsets. (d) Impact of different data proportions on model performance. The top-left section summarizes the trends of Meta-Mol across three datasets under various influencing factors, considering the PR-AUC metric. The other three radial sections compare the performance of different methods within the Tox21 dataset. (e) ROC-AUC performance across different shots. (f) Comparison of ROC-AUC and PR-AUC for various models on the MUV dataset.

Additionally, we evaluated the performance of other representative methods on the Tox21 and SIDER datasets with 10-shot tasks. These methods include multi-task-based approaches such as Weave [19], Attentive FP [18], and CMPNN [17] and pretrained graph neural network models like MAT [15], CDDD [14], MolPMoFiT [13], and N-Gram [51], as well as other approaches including GAT [52], TrimNet [53], and Mol2Context-vec [54]. The results are shown in Fig. 3a, where Meta-Mol demonstrates competitive performance on both datasets. Notably, on the SIDER dataset, Meta-Mol outperforms Mol2Context-vec by ~10%. The loss curves for benchmark datasets including Tox21, SIDER, PCBA, and MUV are presented in Fig. 3b, indicating the effectiveness of the training process across different datasets. For the ToxCast dataset, we evaluated the performance of Meta-Mol and other benchmark models on nine subsets in both 1-shot and 10-shot scenarios, and the results are shown in Fig. 3c. Meta-Mol achieved significant improvements across all tasks, consistently outperforming the benchmarks. By implementing task-specific posteriors through Bayesian methods and a hypernetwork, Meta-Mol enhances its ability to adapt to new tasks. This guides the meta-learning parameters more effectively while preventing overfitting.

### Effects of sample quantity and proportion on Meta-Mol

In real-world drug design scenarios, data are often scarce and imbalanced. Understanding how the model adapts to different levels of data availability and imbalance is highly intriguing. First, we evaluated the performance of Meta-Mol on five datasets with varying amounts of data. The results, as shown in Fig. 3e, illustrate how the model performs under different sample sizes (1-shot, 3-shot, 5-shot, and 10-shot). As the number of samples increases, the ROC-AUC scores generally improve, which is intuitive. However, for Meta-Mol, the changes in ROC-AUC scores across the five datasets are not as significant, and the model consistently achieves good results. This stability can be attributed to Meta-Mol’s probabilistic optimization framework with effective molecular representation, which allows it to maintain high performance even with limited data. Additionally, the effective molecular representation enables the model to accurately generalize the meta-knowledge of properties and structures, thereby achieving better adaptability across various datasets. The results indicate that while Meta-Mol can achieve high performance with limited data, the availability of more samples generally enhances the model’s predictive accuracy, especially in cases of extreme data scarcity, such as with the MUV dataset, where performance improved by 3% from 1-shot to 10-shot. This suggests that even a slight increase in dataset size can significantly improve the model’s performance. To visually demonstrate Meta-Mol’s advantages on the particularly challenging dataset (MUV dataset), we also compared its ROC-AUC and PR-AUC performance against other baseline models. As shown in Fig. 3f, even on the MUV dataset, which suffers from severe label loss, Meta-Mol’s performance comprehensively surpassed other methods, further highlighting its robustness in scenarios with low data and potential label noise.

Then, we further evaluated the performance of Meta-Mol across different sample proportions. The results, as shown in Fig. 3d, indicate the model’s ROC-AUC and PR-AUC scores for multiple datasets under various conditions of sample proportions. The results demonstrate that Meta-Mol consistently outperforms other models (RF, Graph Conv, Siamese Net, MatchingNet, PrototypicalNet) across different sample proportions. For instance, under the 5+/10− condition, Meta-Mol achieves an ROC-AUC of 0.849 ± 0.004 and a PR-AUC of 0.771 ± 0.008 for the Tox21 dataset. Similarly, under the 1+/10− condition, the model’s ROC-AUC improves to 0.879 ± 0.005 with a PR-AUC of 0.580 ± 0.004. Notably, across different sample proportion settings, Meta-Mol consistently shows significant improvements in PR-AUC, with an increase of ~40%. These results suggest that Meta-Mol can generalize well across different sample proportions, highlighting its robustness and adaptability. In conclusion, Meta-Mol’s robustness and adaptability make it a highly promising tool for practical drug design. Its proficiency in handling scarce and imbalanced data can significantly accelerate and improve the drug discovery process.

### A closer look at Meta-Mol

#### Ablation study of Meta-Mol

**Figure 4:**
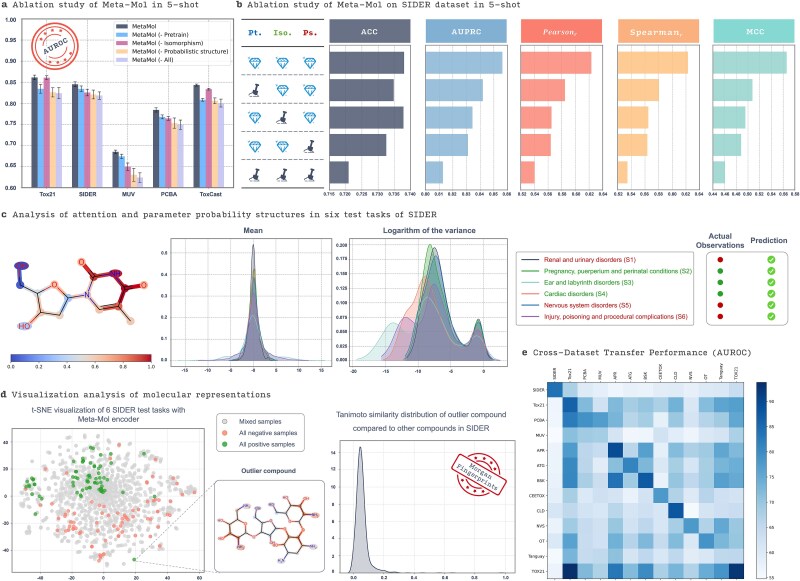
A closer look at Meta-Mol. (a) Ablation study of Meta-Mol in 5-shot evaluation across five datasets. (b) Refined ablation study of Meta-Mol in a 5-shot scenario on the SIDER dataset. (c) Analysis of attention and parameter probability structures across six SIDER test tasks. Highlighted markers indicate whether the experimental result for a molecule in a given task is positive (1) or negative (0). (d) Visual analysis of molecular representations. The outputs of the Meta-Mol encoder are used as molecular representations, which are reduced in dimensionality using t-SNE for visualization. Different marker styles distinguish molecules with mixed, all-negative, or all-positive labels across the six test tasks. (e) Visual analysis of Meta-Mol’s performance (AUROC) across datasets. The rows represent source datasets used for training, and columns represent target datasets for fine-tuning. Full names for ToxCast subsets (e.g. ATG, BSK, CLD) are detailed in Table S4.

To thoroughly evaluate the contribution of key components within the Meta-Mol framework, we conducted a comprehensive ablation study using the 5-shot learning scenario across multiple benchmark datasets, including Tox21, SIDER, MUV, PCBA, and ToxCast (Fig. 4a). The full Meta-Mol model, which incorporates pretraining, an isomorphism-based encoder, and a Bayesian probabilistic structure, consistently outperformed all ablated versions. Specifically, removing the pretraining component led to a notable decrease in performance, particularly on datasets such as SIDER and Tox21, underscoring the critical role of pretraining in enhancing the model’s generalization ability. The results further indicate that the GIN, by better recognizing and preserving the topological information of molecular graphs, excels in tasks requiring detailed structural information—often a critical aspect of molecular representation. Additionally, integrating a Bayesian probabilistic structure (see Materials and Methods) improved the model’s robustness by effectively handling uncertainty, as evidenced by the superior performance of the complete Meta-Mol model.

In the 5-shot evaluation on the SIDER dataset (Fig. 4b), a refined ablation study provided deeper insights into how different components contribute to various performance metrics, including PR-AUC, Pearson correlation, Spearman correlation, and Matthews Correlation Coefficient (MCC). The full Meta-Mol model exhibited the highest scores across all metrics, with the probabilistic structure playing a pivotal role in enhancing the model’s predictive power and consistency. This analysis confirms that each component of Meta-Mol is indispensable, collectively enabling the model to achieve state-of-the-art performance in molecular property prediction tasks.

#### Visual analysis of molecular representation and task adaptation in Meta-Mol

To delve deeper into how Meta-Mol handles molecular representation and task adaptation, we conducted a series of detailed visual analyses. These analyses provide insight into the model’s ability to capture complex molecular structures and its effectiveness in adapting to various predictive tasks, particularly within the challenging SIDER dataset.

We selected a molecule [e.g. CHEMBL6497, “CC1 = CN(C(=O)NC1 = O)C2CC(C(O2)CO)O”] from the SIDER dataset that the model predicted with high accuracy across multiple tasks. This molecule exhibited three positive and three negative labels across six distinct tasks, as shown in Fig. 4c (right). To visualize how Meta-Mol’s encoder focuses on different parts of the molecule, we utilized attention weight visualization. Specifically, we modified the encoder’s pooling layer to use *GlobalAttention* method, which allows for a more nuanced representation of molecular substructures. The attention map (Fig. 4c, left) clearly indicates that the encoder assigns higher attention weights to the pyrimidine ring derivatives within the molecule. This substructure, particularly the pyrimidinedione moiety, is structurally similar to the core of barbiturates, a class of compounds known for their central nervous system depressant effects. The model’s ability to focus on such pharmacologically relevant substructures underscores the encoder’s capability to generalize molecular features effectively, making it well suited for downstream tasks with diverse requirements. Furthermore, the middle panels of Fig. 4c illustrate the distribution of the mean and log-variance of the high-dimensional parameters output by the hypernetwork for the classifier module across the six SIDER tasks. These distributions reveal that despite using a consistent encoder, Meta-Mol learns distinct Gaussian distributions for each task, thereby achieving task-specific adaptation. This capability is critical for handling the heterogeneity inherent in multi-task molecular prediction, as it allows the model to tailor its predictions to the unique characteristics of each task.

In addition to attention mechanisms, we explored how Meta-Mol’s encoder represents molecules across the six SIDER tasks by utilizing t-distributed Stochastic Neighbor Embedding (t-SNE) to visualize the molecular embeddings (Fig. 4d, left). The embeddings are color-coded based on their labels: “All positive,” “All negative,” and “Mixed,” indicating molecules with entirely positive, entirely negative, or a combination of labels across tasks, respectively. The t-SNE plot demonstrates that Meta-Mol effectively captures both structural and task-specific information. Molecules with consistent labels (all positive or all negative) are well clustered, reflecting the encoder’s success in distinguishing between these molecular properties. Notably, molecules with mixed labels are more evenly distributed, suggesting that the encoder is designed to facilitate rapid adaptation in downstream tasks, thereby requiring fewer training steps to achieve optimal performance. Moreover, we identified an outlier positive sample within the t-SNE plot. To further investigate this, we computed the Tanimoto similarity between the Morgan fingerprints of this outlier and the rest of the dataset (Fig. 4d, right). The resulting similarity distribution shows a significant divergence, explaining the outlier status. This finding underscores Meta-Mol’s sensitivity to molecular diversity, which is essential for robust drug discovery applications.

#### Cross-dataset performance evaluation

To thoroughly assess the generalization capability of Meta-Mol across diverse molecular datasets, we took the initiative to conduct cross-dataset transfer experiments, which have rarely been carried out in previous studies. In these experiments, the model was trained on a source dataset (rows in Fig. 4e) and then fine-tuned on a target dataset with minimal data (columns in Fig. 4e). This setup closely mimics real-world drug discovery scenarios, where data scarcity often forces researchers to leverage knowledge from related tasks or domains.

The results, visualized as a heatmap in Fig. 4e and detailed in the provided figure ((including subsets such as ATG, BSK, SIDER, and Tox21), demonstrate distinct patterns in Meta-Mol’s cross-dataset performance. For instance, when transferring between SIDER and Tox21 datasets, Meta-Mol achieved an average AUROC score of 84.03% (from SIDER to Tox21) and 86.21% (from Tox21 to SIDER) in the few-shot fine-tuning process. These high scores indicate that pretrained molecular representations and meta-knowledge effectively transfer to related tasks, even with limited fine-tuning data. This success can be attributed to Meta-Mol’s ability to efficiently extract general meta-knowledge. Such an ability enables the model to rapidly and effectively adapt to new domain-specific tasks, even when the number of available samples is limited. Conversely, performance declines significantly when the source and target datasets exhibit substantial domain divergence. When transferring from Tox21 to some ToxCast subsets (such as the CLD subset), Meta-Mol’s AUROC score dropped to 65.44%. These results highlight a critical limitation: while Meta-Mol excels in exploiting task similarity, it struggles with datasets that differ in underlying molecular distributions or property labels. This challenge is likely due to the reduced overlap in structural motifs and task-specific inductive biases, which hinder the hypernetwork’s ability to dynamically adjust parameters for entirely new scenarios.

These results highlight the importance of dataset similarity in meta-learning for molecular prediction. Practical applications should prioritize transfer learning between related domains. Future work could explore meta-learning strategies that model inter-dataset relationships and integrate self-supervised pretraining on diverse datasets to enhance cross-domain generalization.

## Conclusion

In this work, we introduced Meta-Mol, a task-adaptive few-shot learning framework leveraging Bayesian MAML, designed to address the challenges of molecular property prediction in low-data scenarios. Meta-Mol integrates an advanced GIN to accurately capture molecular structural features, leveraging both atomic and bond-level information. To further refine model adaptability and mitigate overfitting, a hypernetwork is employed to dynamically adjust task-specific weights within a probabilistic framework. This methodology not only enhances the model’s robustness against overfitting but also significantly improves its capacity to generalize across diverse molecular tasks.

Notably, the performance of Meta-Mol on several benchmark datasets demonstrates its superiority over existing methods, particularly in handling the complexities of molecular property prediction with limited data. By adopting a probabilistic framework for weight adaptation and utilizing a hypernetwork to manage task-specific uncertainties, Meta-Mol achieves a nuanced balance between flexibility and precision. This enables the model to adapt quickly and accurately to new tasks, providing a practical solution for drug discovery processes where data are often scarce and heterogeneous. Furthermore, Meta-Mol’s ability to generalize molecular knowledge across different tasks highlights its potential for broader applications in computational chemistry and drug design. The framework’s underlying principles could be extended beyond molecular property prediction to other domains where data scarcity and task variability are significant challenges.

However, while Meta-Mol demonstrates strong performance, certain limitations should be acknowledged. The framework’s reliance on task similarity for effective meta-knowledge transfer means its performance can degrade when meta-training and meta-testing tasks are highly dissimilar, as observed in some cross-dataset experiments. Although the Bayesian framework and hypernetwork aim to mitigate overfitting, extreme data scarcity (e.g. very few tasks for meta-training or extremely low-shot scenarios beyond those tested) could still challenge the model’s ability to learn a robust meta-prior. Therefore, opportunities remain for the further enhancement of Meta-Mol. For instance, the sampling strategy from the task pool could be refined by learning the correlations between tasks, perhaps by employing graph-based methods to model task relationships or using reinforcement learning to optimize the sampling policy, thereby enabling more targeted training and improving the model’s ability to extract meta-knowledge. Additionally, ensuring the stability of the hypernetwork-based model remains a challenging and crucial area for improvement, which could be addressed by exploring regularization techniques specific to hypernetworks or investigating more robust architectures for generating weight parameters. Future work will explore the potential of Meta-Mol for practical applications, such as protein mutation prediction and kinase activity forecasting, and will focus on extending its adaptability to regression tasks.

In summary, Meta-Mol sets a new standard in few-shot molecular property prediction by combining cutting-edge techniques in meta-learning and Bayesian inference. While already demonstrating state-of-the-art performance, the framework’s flexibility and adaptability suggest that further innovations could yield even greater advances in the field.

**Key Points**

- Meta-Mol establishes a Bayesian meta-learning paradigm specifically for low-data drug discovery. By learning a probabilistic distribution over model parameters for each task, it effectively quantifies prediction uncertainty and mitigates overfitting, which is critical for robust performance in sparse and noisy data regimes characteristic of molecular property prediction.
- A core innovation is the use of a hypernetwork to replace the conventional gradient-based inner-loop optimization of MAML. This hypernetwork dynamically generates the parameters for the task-specific posterior weight distribution, enabling more flexible, nongradient-based adaptation and allowing the model to capture more complex relationships between molecular structures and their properties.
- Leveraging an advanced atom-bond graph isomorphism network for superior molecular feature extraction, the complete Meta-Mol framework achieves state-of-the-art performance. It significantly outperforms existing methods across a comprehensive suite of benchmarks, including Tox21, SIDER, MUV, PCBA, and ToxCast, thereby validating its efficacy and strong generalization capability in few-shot learning scenarios.

## Supplementary Material

Supplementary_information_bbaf408

Table_S1_bbaf408

Table_S2_bbaf408

Table_S3_bbaf408

Table_S4_bbaf408

Table_S5_bbaf408

## Data Availability

The evaluation datasets of this study were extracted from MoleculeNet benchmark: https://moleculenet.org/datasets. All codes of Meta-Mol can be found on GitHub (https://github.com/antwiser/Meta-Mol).
