# Supplementary material for: Pushing the boundaries of few-shot learning for low-data drug discovery with a Bayesian meta-learning hypernetwork framework
Source: Brief Bioinform. 2025 Aug 15;26(4):bbaf408. doi: 10.1093/bib/bbaf408 (PMC12354953; doi:10.1093/bib/bbaf408)
Supplement: Supplementary_information_bbaf408 [file supplementary_information_bbaf408.docx]

**Pushing the Boundaries of Few-shot Learning for Low-Data Drug Discovery with a Bayesian Meta-learning Hypernetwork Framework**

**Table of content**

1. [Supplementary Notes 2](#_Toc193873730)

[Note 1. Datasets description and splitting 2](#_Toc193873731)

1. [Supplementary Tables 3](#_Toc193873732)

[Table S1. Dataset statistics. 3](#_Toc193873733)

[Table S2. Details of sub-datasets of ToxCast. 3](#_Toc193873734)

[Table S3. Coded information for atomic and bond features. 3](#_Toc193873735)

[Table S4. The key hyper-parameters of Meta-Mol. 4](#_Toc193873736)

[Table S5. Pseudocode of Meta-Mol. 4](#_Toc193873737)

# Supplementary Notes

## Note 1. Datasets description and splitting

The Tox21 dataset contains 7,831 compounds and 12 tasks, where each task represents the toxicological activity of the compounds against different targets. For example, the task “NR-ER” includes endocrine-disrupting chemicals that interact with nuclear receptors (NR), such as the estrogen receptor (ER). The SIDER dataset comprises 1,427 compounds and 27 tasks corresponding to 27 body organs, with each task representing an adverse reaction that a marketed drug may cause to an organ. The MUV dataset includes 93,127 compounds and 17 tasks, where each task represents a different biological activity. The ToxCast dataset contains 8,615 compounds and 617 tasks, with each task representing a distinct biological mechanism and toxicity correlation. Finally, the PCBA dataset includes 437,929 compounds and 128 tasks, consisting of small molecule bioactivity data generated through high-throughput screening. The splits of each dataset are provided in **Table S1**.

# Supplementary Tables

## Table S1. Dataset statistics.

| **Dataset** | **Tox21** | **SIDER** | **MUV** | **ToxCast** | **PCBA** |
| --- | --- | --- | --- | --- | --- |
| Compound | 7831 | 1427 | 93127 | 8575 | 437929 |
| Task | 12 | 27 | 17 | 617 | 128 |
| Meta-train task | 9 | 21 | 12 | 451 | 118 |
| Meta-test task | 3 | 6 | 5 | 158 | 10 |
| Label active (%) | 6.24 | 56.76 | 0.31 | 12.60 | 0.84 |
| Label inactive (%) | 76.71 | 43.24 | 15.76 | 72.43 | 59.84 |
| Missing Label (%) | 17.05 | 0 | 84.21 | 14.97 | 39.32 |

## Table S2. Details of sub-datasets of ToxCast.

| **Assay Provider** | **APR** | **ATG** | **BSK** | **CEETOX** | **CLD** | **NVS** | **OT** | **TOX21** | **Tanguay** |
| --- | --- | --- | --- | --- | --- | --- | --- | --- | --- |
| Compound | 1039 | 3423 | 1445 | 508 | 305 | 2130 | 1782 | 8241 | 1039 |
| Property | 43 | 146 | 115 | 14 | 19 | 139 | 15 | 100 | 18 |
| Meta-train task | 33 | 106 | 84 | 10 | 14 | 100 | 11 | 80 | 13 |
| Meta-test task | 10 | 40 | 31 | 4 | 5 | 39 | 4 | 20 | 5 |
| Label active (%) | 10.30 | 5.92 | 17.71 | 22.26 | 30.72 | 3.21 | 9.78 | 5.39 | 8.05 |
| Label inactive (%) | 61.61 | 93.92 | 82.29 | 76.38 | 68.30 | 4.52 | 87.78 | 86.26 | 90.84 |
| Missing Label (%) | 28.09 | 0.16 | 0 | 1.36 | 0.98 | 92.27 | 2.44 | 8.35 | 1.11 |

## Table S3. Coded information for atomic and bond features.

| **Type** | **Feature name** | **Range** | **Description** |
| --- | --- | --- | --- |
| Atom feature | atom symbol | 118 | Atomic number (integer ranging from 1 to 118) |
|  | chirality type | 4 | Chirality type including unspecified, tetrahedral (clockwise or counterclockwise), and others |
| Bond feature | bond type | 4 | Bond type including single, double, triple, and aromatic bonds |
|  | bond direction | 3 | Bond direction for double bonds, indicating the spatial orientation such as no direction, upward, or downward |

## Table S4. The key hyper-parameters of Meta-Mol.

| **Hyper-parameter** | **Description** | **Selected** |
| --- | --- | --- |
| *lr* | optimizer’s learning rate for final loss | 1e-3 |
| *hn_head_len* | hypernetwork depth | 3 |
| *hn_hidden_size* | hypernetwork width | 256 |
| *stop_epoch* | epochs | 2500 |
| *milestones* | milestones for MultiStepLR | 101, 1100 |
| *kl_stop_val* | final value of $\gamma$, a parameter that controls the weight of the KL divergence term | 1e-5 |
| *hn_sup_aggregation* | the method for aggregating support samples from the same class in the model | mean |
| *hm_weight_set_num_train* | number of randomly generated weights for training | 5 |

## Table S5. Pseudocode of Meta-Mol.

| **Algorithm 1** Pseudocode of Meta-Mol for molecular property prediction |
| --- |
| **Require:** A set of tasks for predicting molecular properties $T$; |
| **Ensure:** Meta-Mol parameters $\theta$, set step size $\alpha$, $lr$  Randomly initialize $\theta$;  **while** not done **do**  Sample batch of tasks $T_{train}\sim T$ |
| **for all** $T_{train}$ **do** |
| Sample support set $D_{train}^{S}$ and query set $D_{train}^{Q}$ from $T_{train}$ |
| Compute support embeddings $E_{S}$:  $E_{S}\leftarrow E(D_{train}^{S})$  Concatenate $E_{S}$ with true labels $Y_{S}$ and predicted labels $\hat{Y_{S}}$:  $E_{S}^{'}\leftarrow concat(E_{S},Y_{S},\hat{Y_{S}})$ |
| Compute adapted parameters with gradient descent and hypernetwork, weighted by the warm-up coefficient $p$: |
| $\theta_{i}^{'}\leftarrow\theta-\alpha p\nabla_{\theta}\mathcal{L}\left( f_{\theta}\left( D_{i}^{s} \right) \right)+(1-p)\Delta\theta_{i}$  Evaluate $\mathcal{L}_{ce}\mathcal{=L(}f_{\theta_{i}^{'}}(D_{i}^{Q}))$  Evaluate $\mathcal{L}_{KL}=\gamma KL(q(\theta_{i}^{'}\mathcal{)\parallel N(}\theta_{i}^{'}\vert0,I))$  Compute $\mathcal{L}_{T_{train}}^{'}=\mathcal{L}_{ce}+\mathcal{L}_{KL}$  **end for**  Update $\theta\leftarrow\theta- lr\nabla_{\theta}\sum_{T_{train}\sim p(T)} \mathcal{L}_{T_{train}}^{'}$  **end while**  Sample batch of tasks $T_{test}\sim T$  **for all** $T_{test}$ **do**  Sample support set $D_{test}^{S}=\{D_{1},D_{2},\ldots,D_{K}\}\in D_{test}$ and query set $D_{test}^{Q}$ from $T_{test}$  // Similar to the training phase  Compute $\theta_{\text{test}}^{'}$ with gradient descent and hypernetwork  Evaluate the final predictions on the query set $D_{test}^{Q}$:  $y_{\text{test}}^{Q}\leftarrow f_{\theta_{\text{test}}^{'}}(D_{\text{test}}^{Q})$  **end for** |
