# Supplementary material for: Pushing the boundaries of few-shot learning for low-data drug discovery with a Bayesian meta-learning hypernetwork framework
Source: Brief Bioinform. 2025 Aug 15;26(4):bbaf408. doi: 10.1093/bib/bbaf408 (PMC12354953; doi:10.1093/bib/bbaf408)
Supplement: Table_S1_bbaf408 [file table_s1_bbaf408.docx]

**Table S1.** Coded information for atomic and bond features.

| **Type** | **Feature name** | **Range** | **Description** |
| --- | --- | --- | --- |
| Atom feature | atom symbol | 118 | Atomic number (integer ranging from 1 to 118) |
|  | chirality type | 4 | Chirality type including unspecified, tetrahedral (clockwise or counterclockwise), and others |
| Bond feature | bond type | 4 | Bond type including single, double, triple, and aromatic bonds |
|  | bond direction | 3 | Bond direction for double bonds, indicating the spatial orientation such as no direction, upward, or downward |
