# Supplementary material for: Pushing the boundaries of few-shot learning for low-data drug discovery with a Bayesian meta-learning hypernetwork framework
Source: Brief Bioinform. 2025 Aug 15;26(4):bbaf408. doi: 10.1093/bib/bbaf408 (PMC12354953; doi:10.1093/bib/bbaf408)
Supplement: Table_S2_bbaf408 [file table_s2_bbaf408.docx]

**Table S2.** Pseudocode of Meta-Mol.

| **Algorithm 1** Pseudocode of Meta-Mol for molecular property prediction |
| --- |
| **Require:** A set of tasks for predicting molecular properties $T$, Encoder $E(*)$ |
| **Ensure:** Meta-Mol parameters $\theta$, set step size $\alpha$, $lr$, KL weight $\gamma$, warm-up coefficient $p$  Randomly initialize $\theta$;  **while** not done **do**  Sample batch of tasks $T_{train}\sim T$ |
| **for all** $T_{train}$ **do** |
| Sample support set $D_{train}^{S}$ and query set $D_{train}^{Q}$ from $T_{train}$  Compute support embeddings $E_{S}$: $E_{S}\leftarrow E(D_{train}^{S})$  Compute predictions on support set: $\hat{Y_{S}}\leftarrow f_{\theta}(E_{S})$  Form hypernetwork input: $E_{S}^{'}\leftarrow concat(E_{S},Y_{S},\hat{Y_{S}})$  Get posterior parameters from hypernetwork: $\mu, logvar\leftarrow H_{\Omega}(E_{S}^{'})$ |
| Compute inner-loop adapted parameters with gradient descent and hypernetwork, weighted by the warm-up coefficient $p$: |
| 1. calculate support set loss: ${\mathcal{\mathcal{L}}}_{support}=f_{\theta}\left( D_{i}^{s} \right)$  2. calculate KL loss: ${\mathcal{\mathcal{L}}}_{kl}=\lambda KL(\mathcal{N(}\mu, exp(logvar))\mathcal{\parallel N(}0,I))$  3. add KL divergence regularization: ${\mathcal{\mathcal{L}}}_{support}={\mathcal{\mathcal{L}}}_{support}+{\mathcal{\mathcal{L}}}_{kl}$  4. update parameters:  $\mu_{i}^{'}\leftarrow\left( 1-p \right)\Delta\mu_{i}+\alpha p\nabla_{\theta}{\mathcal{\mathcal{L}}}_{support}$  $\theta_{i}^{'}\leftarrow\mu_{i}^{'}+\exp\left( \frac{1}{2}\mathrm{logvar}_{i} \right)⨀\epsilon$ (Reparameterization)  Calculate query set loss and outer-loop parameters:  ${\mathcal{\mathcal{L}}}_{T_{train}}^{'}\mathcal{=\mathcal{L}}\left( f_{\theta_{i}^{'}}\left( D_{i}^{Q} \right) \right)+\gamma KL(q(\theta_{i}^{'}\mathcal{)\parallel N(}\theta_{i}^{'}\vert0,I))$  **end for**  Update $\theta\leftarrow\theta- lr\nabla_{\theta}\sum_{T_{train}\sim p(T)} {\mathcal{\mathcal{L}}}_{T_{train}}^{'}$  **end while**  Sample batch of tasks $T_{test}\sim T$  **for all** $T_{test}$ **do**  Sample support set $D_{test}^{S}=\{D_{1},D_{2},\ldots,D_{K}\}\in D_{test}$ and query set $D_{test}^{Q}$ from $T_{test}$  // Similar to the training phase  Compute $\theta_{\text{test}}^{'}$ with gradient descent and hypernetwork  Evaluate the final predictions on the query set $D_{test}^{Q}$:  $y_{\text{test}}^{Q}\leftarrow f_{\theta_{\text{test}}^{'}}(D_{\text{test}}^{Q})$  **end for** |
