# Supplementary material for: Pushing the boundaries of few-shot learning for low-data drug discovery with a Bayesian meta-learning hypernetwork framework
Source: Brief Bioinform. 2025 Aug 15;26(4):bbaf408. doi: 10.1093/bib/bbaf408 (PMC12354953; doi:10.1093/bib/bbaf408)
Supplement: Table_S3_bbaf408 [file table_s3_bbaf408.docx]

**Table S3**. Dataset statistics.

| **Dataset** | **Tox21** | **SIDER** | **MUV** | **ToxCast** | **PCBA** |
| --- | --- | --- | --- | --- | --- |
| Compound | 7831 | 1427 | 93127 | 8575 | 437929 |
| Task | 12 | 27 | 17 | 617 | 128 |
| Meta-train task | 9 | 21 | 12 | 451 | 118 |
| Meta-test task | 3 | 6 | 5 | 158 | 10 |
| Label active (%) | 6.24 | 56.76 | 0.31 | 12.60 | 0.84 |
| Label inactive (%) | 76.71 | 43.24 | 15.76 | 72.43 | 59.84 |
| Missing Label (%) | 17.05 | 0 | 84.21 | 14.97 | 39.32 |
