# Supplementary material for: Pushing the boundaries of few-shot learning for low-data drug discovery with a Bayesian meta-learning hypernetwork framework
Source: Brief Bioinform. 2025 Aug 15;26(4):bbaf408. doi: 10.1093/bib/bbaf408 (PMC12354953; doi:10.1093/bib/bbaf408)
Supplement: Table_S4_bbaf408 [file table_s4_bbaf408.docx]

**Table S4.** Details of sub-datasets of ToxCast.

| **Assay Provider** | **APR** | **ATG** | **BSK** | **CEETOX** | **CLD** | **NVS** | **OT** | **TOX21** | **Tanguay** |
| --- | --- | --- | --- | --- | --- | --- | --- | --- | --- |
| Compound | 1039 | 3423 | 1445 | 508 | 305 | 2130 | 1782 | 8241 | 1039 |
| Property | 43 | 146 | 115 | 14 | 19 | 139 | 15 | 100 | 18 |
| Meta-train task | 33 | 106 | 84 | 10 | 14 | 100 | 11 | 80 | 13 |
| Meta-test task | 10 | 40 | 31 | 4 | 5 | 39 | 4 | 20 | 5 |
| Label active (%) | 10.30 | 5.92 | 17.71 | 22.26 | 30.72 | 3.21 | 9.78 | 5.39 | 8.05 |
| Label inactive (%) | 61.61 | 93.92 | 82.29 | 76.38 | 68.30 | 4.52 | 87.78 | 86.26 | 90.84 |
| Missing Label (%) | 28.09 | 0.16 | 0 | 1.36 | 0.98 | 92.27 | 2.44 | 8.35 | 1.11 |
