# Supplementary material for: Pushing the boundaries of few-shot learning for low-data drug discovery with a Bayesian meta-learning hypernetwork framework
Source: Brief Bioinform. 2025 Aug 15;26(4):bbaf408. doi: 10.1093/bib/bbaf408 (PMC12354953; doi:10.1093/bib/bbaf408)
Supplement: Table_S5_bbaf408 [file table_s5_bbaf408.docx]

**Table S5.** The key hyper-parameters of Meta-Mol.

| **Hyper-parameter** | **Description** | **Selected** |
| --- | --- | --- |
| *lr* | optimizer’s learning rate for final loss | 1e-3 |
| *hn_head_len* | hypernetwork depth | 3 |
| *hn_hidden_size* | hypernetwork width | 256 |
| *stop_epoch* | epochs | 2500 |
| *milestones* | milestones for MultiStepLR | 101, 1100 |
| *kl_stop_val* | final value of $\gamma$, a parameter that controls the weight of the KL divergence term | 1e-5 |
| *hn_sup_aggregation* | the method for aggregating support samples from the same class in the model | mean |
| *hm_weight_set_num_train* | number of randomly generated weights for training | 5 |
